# New Lineage of Lassa Virus, Togo, 2016

**DOI:** 10.3201/eid2403.171905

**Published:** 2018-03

**Authors:** Shannon L.M. Whitmer, Thomas Strecker, Daniel Cadar, Hans-Peter Dienes, Kelly Faber, Ketan Patel, Shelley M. Brown, William G. Davis, John D. Klena, Pierre E. Rollin, Jonas Schmidt-Chanasit, Elisabeth Fichet-Calvet, Bernd Noack, Petra Emmerich, Toni Rieger, Svenja Wolff, Sarah Katharina Fehling, Markus Eickmann, Jan Philipp Mengel, Tilman Schultze, Torsten Hain, William Ampofo, Kofi Bonney, Juliana Naa Dedei Aryeequaye, Bruce Ribner, Jay B. Varkey, Aneesh K. Mehta, G. Marshall Lyon, Gerrit Kann, Philipp De Leuw, Gundolf Schuettfort, Christoph Stephan, Ulrike Wieland, Jochen W.U. Fries, Matthias Kochanek, Colleen S. Kraft, Timo Wolf, Stuart T. Nichol, Stephan Becker, Ute Ströher, Stephan Günther

**Affiliations:** Centers for Disease Control and Prevention, Atlanta, Georgia, USA (S.L.M. Whitmer, K. Patel, S.M. Brown, W.G. Davis, J.D. Klena, P.E. Rollin, S.T. Nichol, U. Ströher);; Philipps University, Marburg, Germany (T. Strecker, S. Wolff, S.K. Fehling, M. Eickmann, S. Becker);; German Center for Infection Research (DZIF), Partner sites, Hamburg, Giessen, and Marburg, Germany (T. Strecker, S. Wolff, S.K. Fehling, M. Eickmann, T. Hain, S. Becker, S. Günther);; Bernhard Nocht Institute for Tropical Medicine, Hamburg (D. Cadar, J. Schmidt-Chanasit, E. Fichet-Calvet, B. Noack, P. Emmerich, T. Rieger, S. Günther);; University Hospital of Cologne, Cologne, Germany (H.-P. Dienes, U. Wieland, J.W.U. Fries, M. Kochanek);; Hospital of Hope, Mango, Togo (K. Faber); University of Rostock, Rostock, Germany (P. Emmerich);; Justus-Liebig University Giessen, Giessen (J.P. Mengel, T. Schultze, T. Hain);; Noguchi Memorial Institute for Medical Research, Accra, Ghana (W. Ampofo, K. Bonney, J.N.D. Aryeequaye);; Emory University, Atlanta (B. Ribner, J.B. Varkey, A.K. Mehta, G.M. Lyon III, C.S. Kraft);; University Hospital, Frankfurt/Main, Germany (G. Kann, P. De Leuw, G. Schuettfort, C. Stephan, T. Wolf)

**Keywords:** Lassa fever, Lassa virus, arenaviruses Old World, viral hemorrhagic fever, Togo, viruses

## Abstract

We describe a strain of Lassa virus representing a putative new lineage that was isolated from a cluster of human infections with an epidemiologic link to Togo. This finding extends the known range of Lassa virus to Togo.

Lassa virus is endemic to the West Africa countries of Guinea, Sierra Leone, Liberia, Mali, Côte d’Ivoire, and Nigeria (*1**–**3*). The virus causes Lassa fever, a hemorrhagic disease with a case-fatality rate ≈30% in the current hospital setting in West Africa. So far, 4 lineages of Lassa virus are firmly established: lineages I, II, and III circulate in Nigeria, and lineage IV circulates in Guinea, Sierra Leone, Liberia, Mali, and Côte d’Ivoire (*1**–**3*). Recently, strains from Mali and Côte d’Ivoire were proposed to represent a separate lineage V (*4*). The newly discovered Lassa virus strain Kako from *Hylomyscus pamfi* rodents trapped in Nigeria is designated lineage VI for the purpose of this article (*5*).

Lassa virus has not been previously detected in humans or rodents in Togo; therefore, the virus was not considered endemic to this country. We describe a strain of Lassa virus representing a new lineage that was isolated from a cluster of human infections with an epidemiologic link to Togo (Technical Appendix) (*6**,**7*). The clinical courses of the 3 case-patients and medical and public health interventions are described elsewhere (*8**–**10*).

The Lassa virus infections in the index case-patient, secondary case-patient 1, and secondary case-patient 2 were confirmed by laboratory investigations at Bernhard Nocht Institute (Hamburg, Germany); Centers for Disease Control and Prevention (Atlanta, GA, USA); and Philipps University (Marburg, Germany), respectively. The viruses from all 3 patients were isolated in Vero E6 cell culture in the respective Biosafety Level 4 laboratories. Full-length virus sequences were generated directly from clinical specimens, from the isolates, or both using next-generation sequencing technology in combination with Sanger sequencing (sequences deposited into GenBank under accession nos. KU961971, KU961972, LT601601, LT601602, MF990886–MF990889) (Technical Appendix). The sequence from the index case-patient was submitted to GenBank on March 23, 2016, and immediately made publicly available to support the laboratory and public health response in Togo and the other affected countries. 

The virtually identical viruses from the 3 patients confirmed the transmission chains suggested by the epidemiologic data. Only the virus from secondary case-patient 2 showed differences in coding regions—a deletion of 3 nt and a nucleotide exchange in the polymerase (L) gene—from the viruses in the other 2 case-patients. These differences were confirmed by sequencing the virus in the clinical specimens. Differences among the 3 strains in the highly structured intergenic regions might represent artifacts created by the difficulty in sequencing these regions.

The phylogeny was inferred using BEAST2 (https://www.beast2.org/) with nucleotide sequences of full-length nucleoprotein (NP), glycoprotein precursor (GPC), and L and Z genes of the Togo strain in conjunction with representative sequences of Lassa virus and other Old World arenaviruses. The most stable reconstruction was obtained for the L gene with the Togo strain being placed in sister relationship with lineage II (all branches with posterior support values >0.97) (Figure). In the NP- and GPC-based phylogenies, the Togo strain clusters with lineages I and VI (Pinneo and Kako strains); however, the branching order is not well supported (posterior values 0.51–0.86) (Figure). The phylogeny based on the small Z gene further supports a relationship of the Togo strain with lineages I, II, and VI (Technical Appendix Figure 1). The ambiguous position of the Togo strain relative to lineages I, II, and VI is consistent with a recombination analysis showing that most of the L gene sequence is related to lineage II, and NP and GPC comprise sequence stretches mainly related to lineages I and VI (Technical Appendix Figure 2). This mosaic structure might be the result of recombination, reassortment, or both or might have evolved by chance.

**Figure:**
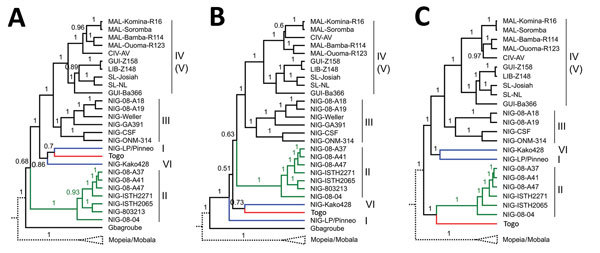
Phylogeny of the Lassa virus strain from Togo, 2016. Phylogenetic trees were inferred using BEAST2 (https://www.beast2.org/) for full-length glycoprotein precursor (A), nucleoprotein (B), and polymerase (C) genes. The analysis included representative Lassa virus strains and other Old World arenaviruses (Technical Appendix). Posterior support values are shown at the branches. Lassa virus lineages are indicated by roman numbers on the right. The branch for Mopeia and Mobala virus is shown schematically and the branches for the remaining Old World arenaviruses have been removed for clarity of presentation. The branches for the Togo strain and most closely related Lassa virus lineages are marked (red, Togo; green, lineage II; blue, lineages I and VI). The origins of the Lassa virus strains are abbreviated as follows: CIV, Côte d’Ivoire; GUI, Guinea; LIB, Liberia; MAL, Mali; NIG, Nigeria; SL, Sierra Leone.

The long branch (i.e., large phylogenetic distance) separating the Togo strain from known lineages suggests that it represents a new lineage. Because Lassa virus lineages were originally established on the basis of uncorrected sequence distances (*1*), we used the same method here. The frequency distribution of pairwise amino acid distances in GPC, NP, and L between the Togo strain and all other Lassa virus strains perfectly overlaps with the distribution of distances between Lassa virus lineages I, II, III, IV, and VI indicating that the Togo strain is a separate lineage (Technical Appendix Table 1). However, we noted that the distance between the proposed lineage V and lineage IV rather corresponds to intralineage distances, and therefore, we considered lineage V a subclade of lineage IV in our analysis (Technical Appendix Table 1). We propose that formal recognition of Lassa virus lineages should be decided by the International Committee on Taxonomy of Viruses.

In conclusion, sequencing Lassa virus from a cluster of imported infections, with the index case-patient originating from Togo, reveals a new lineage of Lassa virus in West Africa. It seems to be related to lineage II or lineages I/VI, which are all circulating in Nigeria.

## About the Author

Technical Appendix Additional methods used to detect a new Lassa virus strain, Togo, 2016; uncorrected pairwise amino acid distances among Old World arenaviruses; phylogeny of the Lassa virus Togo strain using Z gene sequences; and analysis of potential recombination and/or reassortment in its evolution.
