# Supplementary material for: New Lineage of Lassa Virus, Togo, 2016
Source: Emerg Infect Dis. 2018 Mar;24(3):599–602. doi: 10.3201/eid2403.171905 (PMC5823357; doi:10.3201/eid2403.171905)
Supplement: Technical Appendix — Additional methods used to detect a new Lassa virus strain, Togo, 2016; uncorrected pairwise amino acid distances among Old World arenaviruses; phylogeny of the Lassa virus Togo strain using Z gene sequences; and analysis of potential recombination and/or reassortment in its evolution. [file 17-1905-Techapp-s1.pdf]

Article DOI: https://doi.org/10.3201/eid2403.171905

New Lineage of Lassa Virus, Togo, 2016

Technical Appendix

Patients

On February 25, 2016, a healthcare professional who worked in Sansanné-Mango, Savanes Region in northern Togo, was air-evacuated with a diagnosis of severe malaria to the University Hospital Cologne (Cologne, Germany). He died within a few hours after hospital admission. Lassa fever was diagnosed postmortem after the suspicion of hemorrhagic fever had been raised on evaluation of histopathologic findings of the liver. Secondary case-patient 1 was a healthcare professional who treated the index case-patient in Togo. Lassa fever was clinically diagnosed in Togo, and the patient was air-evacuated to Atlanta, Georgia, USA, on March 12, 2016, for medical treatment at Emory University Hospital. Secondary case-patient 2 was a mortician in Germany who prepared the body of the index case-patient for repatriation. He was treated at Frankfurt University Hospital (Frankfurt, Germany). Both secondary case-patients survived.

Methods

Lassa Virus Sequencing in Hamburg, Germany

The sequence of Lassa virus strain Togo from the index case-patient was determined directly from clinical specimens using next-generation sequencing combined with Sanger sequencing technology. Briefly, the serum sample was filtered through a 0.45-µm filter (Merck Millipore, Darmstadt, Germany) to remove cell debris and bacteria and treated with a mixture of DNase and RNase to digest unprotected nucleic acids, including host DNA/RNA. RNA was extracted and converted to double-strand cDNA. Library preparation was performed using Nextera XT DNA Library Preparation Kit (Illumina, San Diego, CA, USA). Sequencing was performed on the Illumina MiSeq platform with 250 base-paired ends and dual barcoding for each library. The paired-end reads generated by MiSeq were trimmed and de novo assembled using Geneious 9 (Biomatters, Auckland, New Zealand). Reads and contigs >100 bp were

subjected to mapping using Lassa virus sequences from GenBank. Complete genome assembly coverage for S segment was obtained except for the intergenic regions. Gaps were filled by Sanger sequencing. The genome sequences were deposited at GenBank under accession nos. KU961971.1 and KU961972.1.

Lassa Virus Sequencing in Atlanta, Georgia, USA

Unbiased next-generation sequencing was performed using Illumina TruSeq stranded total RNA using RNA extracted from blood from secondary case-patient 1 (drawn on March 12, 2016) and a viral isolate generated from the same material. RNA was extracted from 100 μL of blood or tissue culture supernatant using MagMAX Pathogen RNA/DNA isolation kit (Thermo Fisher Scientific, Waltham, MA, USA) and BeadRetriever (Thermo Fisher Scientific). Samples were sequenced by Omega Bioservices, Inc. (Norcross, GA, USA), on an Illumina MiSeq using version 2, 2 × 150 cycle kits. Consensus sequences from the viral isolate were generated using Geneious 9.1.3 (Biomatters, Auckland, New Zealand) by mapping reads to KU961971 and KU961972 with 5-fold iterative remapping. Low-quality reads/bases were filtered to Pred scores of 15, and duplicate reads were removed. We observed 100% genome coverage using a 3-fold cutoff for the L and S segments. Consensus sequences from the clinical specimen were generated using Geneious 9.1.3 by mapping reads to KU961971 and KU961972 with 5-fold iterative remapping. Consensus genomes were generated with no read trimming; duplicate removal and cutoffs were set at 3-fold coverage. We observed 90% coverage for the L segment and 96% coverage for the S segment. Using Sanger sequencing, we increased coverage to 100% for the S segment and 95% for the L segment. The genome sequences were deposited at GenBank under accession nos. MF990886 and MF990888 for the virus in blood; and MF990887 and MF990889 for the viral isolate.

Lassa Virus sequencing in Marburg/Giessen, Germany

Vero E6 cells were inoculated with serum of secondary case-patient 2. Genome sequences were determined from viruses after the first passage. Cell culture supernatant was collected, cellular debris were removed by 2 × centrifugation at 2,500 rpm for 10 min at 4°C and subjected to ultracentrifugation through a 20%-sucrose cushion. Pelleted virions were resuspended in RLT buffer (Qiagen, Hilden, Germany) and RNA was extracted using the RNeasy mini kit (Qiagen) according to the manufacturer's manual. rRNA was depleted using the Ribo-Zero Gold Kit (Epicentre, Madison, WI, USA). A cDNA Library was constructed

according to Illumina's TruSeq total RNA protocol as described earlier (*1*). Library quality control was performed with DNA HS Assay on a 2100 Bioanalyzer (Agilent, Santa Clara, CA, USA). Normalized and pooled libraries were then sequenced on an Illumina MiSeq using v3 chemistry and paired-end sequencing (2 × 301 cycles). After adaptor clipping and quality filtering, reads were mapped against Lassa virus L and S segment references (GenBank accession nos. KU961971.1 and KU961972.1, respectively). The median coverage rates for L and S segments were 145 and 185, respectively. Genome regions with poor coverage, as well as variations from to the sequences of the index case-patient and secondary case-patient 1, were verified by Sanger sequencing of clinical samples. The genome sequences were deposited at the European Nucleotide Archive under accession nos. LT601601 and LT601602 (*2*).

Phylogenetic Analysis

Glycoprotein precursor (GPC), nucleoprotein (NP), polymerase (L), and Z nucleotide sequences of Lassa virus strain Togo (GenBank accession nos. KU961971.1, KU961972.1) were aligned with sequences of Lassa virus strains Josiah (GenBank accession nos. J04324, U63094, NC_004297), NL (AY179173, AY179172), Z148 (AY628205, AY628204), Z158 (AY628201, AY628200), Ba366 (GU830839, GU979513), Komina-R16 (KF478767, KF478760), Soromba (KF478765, KF478762), Bamba-R114 (KF478766, KF478761), Ouoma-R123 (KF478768, KF478764), AV (AF246121, AY179171), NIG08-A18 (GU481070, GU481071), NIG08-A19 (GU481072, GU481073), Weller (AY628206), GA391 (X52400), ONM-314 (KT992423, KT992433), CSF (AF333969, AY179174), NIG08-A37 (GU481074, GU481075), NIG08-A41 (GU481076, GU481077), NIG08-A47 (GU481078, GU481079), ISTH2271-NIG-2012 (KM821986, KM821985), ISTH2065-NIG-2012 (KM821974, KM821973), 803213 (AF181854), NIG08–04 (GU481068, GU481069), LP/Pinneo (AF181853, KM822127), Kako 428 (KT992425, KT992435); and the Old World arenaviruses Gbagroube CIV608 (GU830848), Mopeia AN21366 (M33879), Mopeia AN20410 (AY772170, AY772169), Mopeia Mozambique (DQ328874, DQ328875), Morogoro 3017 (EU914103, EU914104), Mobala Acar3080 (AY342390, DQ328876), Gairo 27421 (KJ855308, KJ855307), Menekre CIV1227 (GU830862), Ippy DakAnB188d (DQ328877, DQ328878), Merino-Walk (GU078660, NC023763), Lujo (FJ952384, FJ952385); and lymphocytic choriomeningitis virus strains WE (M22138, AF004519), Armstrong (M20869, J04331, AY847351), CH-5692 (AF325214, DQ868484), CH-5871 (AF325215), Marseille 12 (DQ286931, DQ286932), MX (EU195888, EU195889,

AJ131281), M1 (AB261991), UBC aggressive (EU480450), Traub (DQ868487, DQ868488), Pasteur (DQ868485, DQ868486), Dandenong (EU136038, EU136039), SN05 (FJ895884), GR01 (FJ895883), CABN (FJ895882), and Y (DQ118959).

Phylogenies were inferred with BEAST2 program (*3*) using the general time-reversible (for GPC, NP, and L) or the Hasegawa–Kishino–Yano (for Z) model of sequence evolution with gamma distribution of among-site nucleotide substitution rate, and 10^7 steps with sampling every 10^3rd step. An initial evaluation revealed minimal rate variation among branches in NP and L gene (median ucld.stdev 0.13–0.15); therefore, these phylogenies were inferred with the strict clock model. We used the relaxed log-normal clock model for GPC and Z, as the rate variation was higher (median ucld.stdev 0.24 and 0.28, respectively). Uncorrected pairwise amino acid distances were calculated in MacVector (MacVector, Apex, NC, USA). Analysis of potential recombination or reassortment was conducted with RDP4 (http://web.cbio.uct.ac.za/~darren/rdp.html) (*4*). The aligned GPC, NP, and L nucleotide sequences were concatemerized in the order "L–GPC–reverse complement of NP" according to the gene arrangement in L and S segments. A full exploratory recombination scan (RDP, GENECONV, Bootscan, MaxChi, Chimaera, SiScan, 3Seq) revealed a potential recombination event in GPC of Lassa strain Togo involving NIG08-A41 as major and LP/Pinneo as minor parent (RDP uncorrected $p = 2.3 \times 10^{-5}$, corrected for multiple tests $p = 0.21$). A manual Bootscan analysis was performed for Lassa strain Togo as potential recombinant and NIG08-A41, Kako 428, and LP/Pinneo as parents using a window size of 500 nt with a step size of 10, UPGMA (unweighted pair group method with arithmetic mean) tree, 100 bootstrap replicates, and F84 model.

References

1. Schultze T, Hilker R, Mannala GK, Gentil K, Weigel M, Farmani N, et al. A detailed view of the intracellular transcriptome of *Listeria monocytogenes* in murine macrophages using RNA-seq. Front Microbiol. 2015;6:1199. PubMed http://dx.doi.org/10.3389/fmicb.2015.01199

2. Wolff S, Schultze T, Fehling SK, Mengel JP, Kann G, Wolf T, et al. Genome sequence of Lassa virus isolated from the first domestically acquired case in Germany. Genome Announc. 2016;4:e00938-16. PubMed http://dx.doi.org/10.1128/genomeA.00938-16

3. Bouckaert R, Heled J, Kühnert D, Vaughan T, Wu CH, Xie D, et al. BEAST 2: a software platform for Bayesian evolutionary analysis. PLOS Comput Biol. 2014;10:e1003537. PubMed http://dx.doi.org/10.1371/journal.pcbi.1003537

4. Martin DP, Murrell B, Golden M, Khoosal A, Muhire B. RDP4: Detection and analysis of recombination patterns in virus genomes. Virus Evol. 2015;1:vev003. PubMed http://dx.doi.org/10.1093/ve/vev003

5. Bowen MD, Rollin PE, Ksiazek TG, Hustad HL, Bausch DG, Demby AH, et al. Genetic diversity among Lassa virus strains. J Virol. 2000;74:6992–7004. PubMed http://dx.doi.org/10.1128/JVI.74.15.6992-7004.2000

6. Manning JT, Forrester N, Paessler S. Lassa virus isolates from Mali and the Ivory Coast represent an emerging fifth lineage. Front Microbiol. 2015;6:1037. PubMed http://dx.doi.org/10.3389/fmicb.2015.01037

7. Olayemi A, Cadar D, Magassouba N, Obadare A, Kourouma F, Oyeyiola A, et al. New hosts of the Lassa virus. Sci Rep. 2016;6:25280. PubMed http://dx.doi.org/10.1038/srep25280

Technical Appendix Table. Uncorrected pairwise amino acid distances among Old World arenaviruses*

Gene/strains and species compared	Percentiles of the frequency distribution of pairwise amino acid distances, %†				
	0	25	50	75	100
GPC					
Lassa virus intralineage‡	0.6	3.1	4.3	5.5	8.2
Lassa virus interlineage§	5.1	6.5	7.1	8.1	11.4
Togo vs. other Lassa virus strains	4.9	6.1	6.5	7.3	10.6
Lassa strains vs. other Old World arenavirus species	9.8	21.8	24.2	29.6	43.5
NP					
Lassa virus intralineage‡	0.5	3.3	5.3	6.3	9.6
Lassa virus interlineage§	7.0	8.8	9.7	10.4	14.6
Togo vs. other Lassa virus strains	7.7	8.9	9.2	9.6	11.6
Lassa strains vs. other Old World arenavirus species	14.1	25.5	27.0	32.0	38.6
L					
Lassa virus intralineage‡	0.9	8.4	13.1	15.8	18.8
Lassa virus interlineage§	19.8	21.3	24.6	25.2	26.0
Togo vs. other Lassa virus strains	22.4	24.8	25.2	25.7	26.9
Lassa strains vs. other Old World arenavirus species	41.2	42.0	43.7	49.0	54.7

*GPC, glycoprotein precursor; L, polymerase; NP, nucleoprotein.
†Lassa virus lineages were previously established on the basis of uncorrected amino acid distances. Interlineage distances ranged from 5.7% to 7.4% in GPC and 9.5% to 12% in NP (*5*).
‡The uncorrected pairwise amino acid distances between the previously proposed lineage V (*6*) (strains AV, Komina-R16, Soromba, Bamba-R114, and Ouoma-R123) and lineage IV (strains Josiah, NL, Z148, Z158, and Ba366) ranged from 4.1% to 7.1% in GPC, 6.0% to 7.4% in NP, and 15.1% to 16.5% in L. Because these ranges fall within the distribution of intralineage distances, the proposed lineage V was considered here a subclade of lineage IV rather than a separate lineage. The corresponding distances were included in the intralineage analysis.
§The uncorrected pairwise amino acid distances between Kako virus (*7*) and other Lassa virus strains ranged from 8.6% to 11.4% in GPC, 11.8% to 14.6% in NP, and 23.8% to 25.7% in L. Because these ranges fall within the distribution of interlineage distances, Kako virus was considered here a separate lineage (tentatively designated VI) of Lassa virus and included in the interlineage analysis.

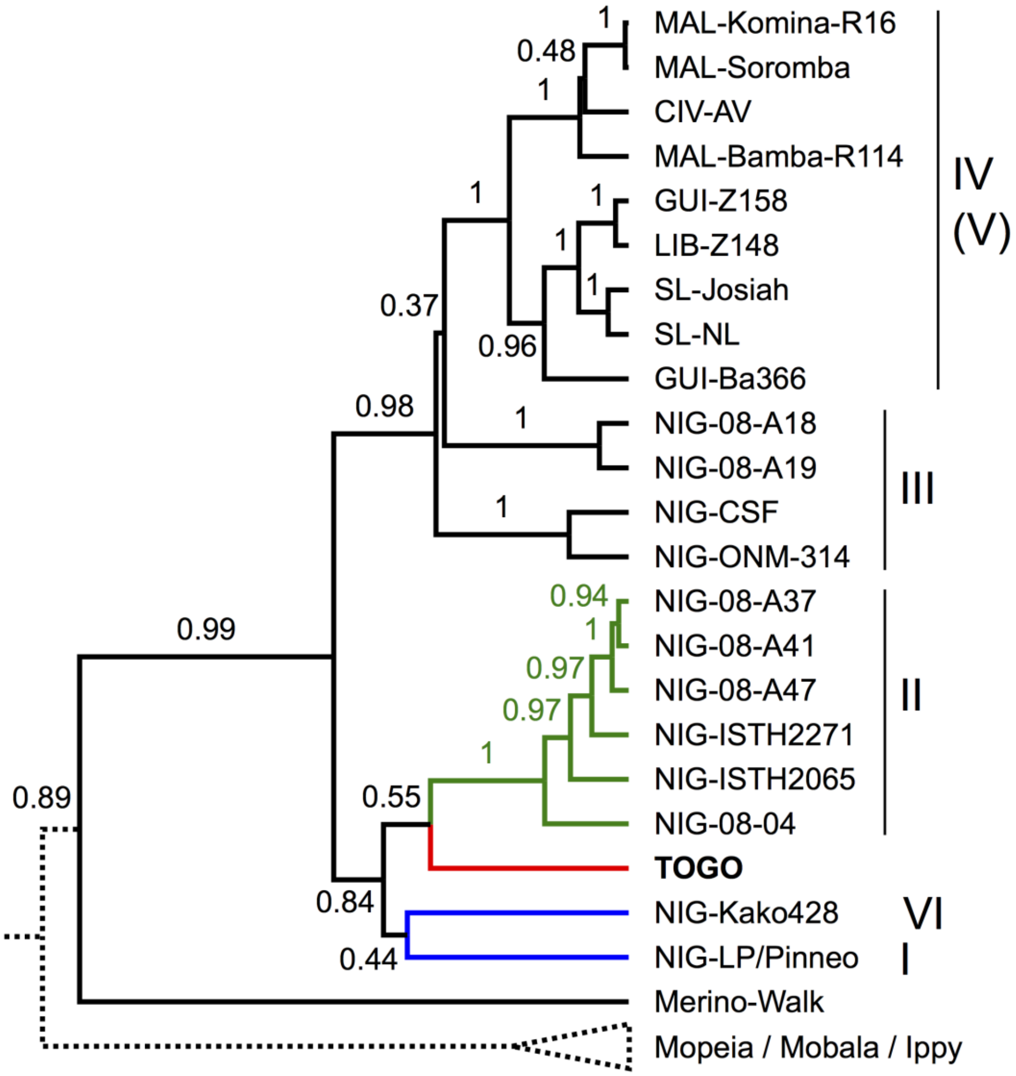

Technical Appendix Figure 1. Phylogeny of the Lassa virus strain from Togo using Z gene sequences. The phylogenetic tree was inferred using BEAST2 program (*6*). Posterior support values are shown at the branches. Lassa virus lineages are indicated by roman numbers on the right. The branch for Mopeia, Mobala, and Ippy viruses is shown schematically and the branches for the remaining Old World arenaviruses have been removed for clarity of presentation. The origin of the Lassa virus strains is abbreviated as follows: CIV, Côte d'Ivoire; GUI, Guinea; LIB, Liberia; MAL, Mali; NIG, Nigeria; SL, Sierra Leone.

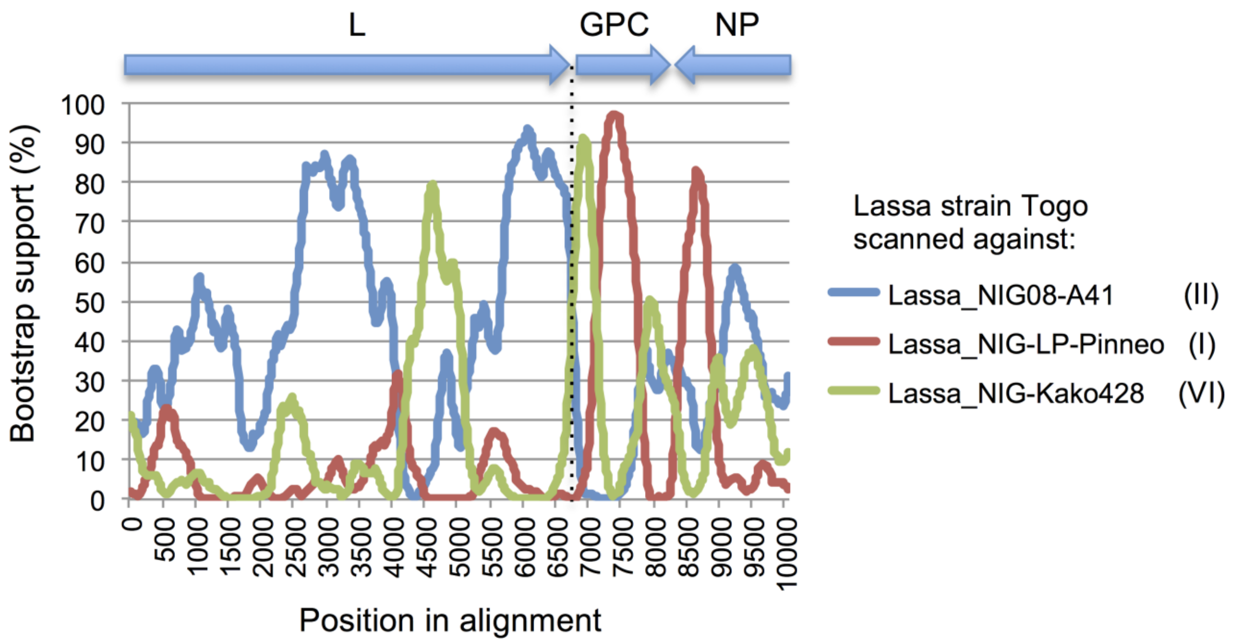

Technical Appendix Figure 2. Analysis of potential recombination and/or reassortment in evolution of Lassa virus strain Togo. A manual Bootscan analysis was performed in RDP4 for Lassa virus strain Togo as potential recombinant and NIG08-A41 (lineage II), Kako 428 (lineage VI), and LP/Pinneo (lineage I) as potential parents. The border between L and S segment is shown by a vertical dotted line. GPC, glycoprotein precursor; L, polymerase; NP, nucleoprotein.
